# Effectiveness of different sampling schemes in predicting adventitious genetically modified maize content in a smallholder farming system

**DOI:** 10.1080/21645698.2020.1846483

**Published:** 2020-12-10

**Authors:** Yun-Syuan Jhong, Wen-Shin Lin, Tien-Joung Yiu, Yuan-Chih Su, Bo-Jein Kuo

**Affiliations:** a Department of Agronomy, National Chung Hsing University, Taichung, Taiwan (R.O.C.); b Department of Plant Industry, National Pingtung University of Science and Technology, Pingtung, Taiwan (R.O.C.); c Tainan District Agricultural Research and Extension Station, Puzi city, COA, Executive Yuan, Taiwan (R.O.C.); d Innovation and Development Center of Sustainable Agriculture (IDCSA), National Chung Hsing University, Taichung, Taiwan (R.O.C.); e Pervasive AI Research (PAIR) Labs, Hsinchu, Taiwan (R.O.C.)

**Keywords:** Genetically modified maize, sampling schemes, pollen dispersal model, smallholder farming system

## Abstract

When genetically modified (GM) maize is planted in an open field, it may cross-pollinate with the nearby non-GM maize under certain airflow conditions. Suitable sampling methods are crucial for tracing adventitious GM content. By using field data and bootstrap simulation, we evaluated the performance of common sampling schemes to determine the adventitious GM content in small maize fields in Taiwan. A pollen dispersal model that considered the effect of field borders, which are common in Asian agricultural landscapes, was used to predict the cross-pollination (CP) rate. For the 2009–1 field data, the six-transect (T_six_), JM method for low expected flow (JM[L]), JM method for high expected flow (JM[H]), and V-shaped transect (T_V_) methods performed comparably to simple random sampling (SRS). T_six_, T_V_, JM(L), and JM(H) required only 13% or less of the sample size required by SRS. After the simulation and verification of the 2009–2 and 2010–1 field data, we concluded that T_six_, T_V_, JM(L), and systematic random sampling methods performed equally as well as SRS in CP rate predictions. Our findings can serve as a reference for monitoring the pollen dispersal tendencies of maize in countries with smallholder farming systems.

## Introduction

Commercial cultivation of genetically modified (GM) crops increases yearly worldwide. GM maize is one of the most common GM crops and the global GM cropping area has increased from 189.8 million hectares in 2017 to 191.7 million hectares in 2018.^1^ Under certain airflow conditions, GM maize planted in an open field may cross-pollinate with non-GM maize in the neighboring fields. Several preventive measures are used to minimize the risk of pollen-mediated gene flow in maize, such as certified seed, asynchronous flowering, barrier zones, isolation distance, and good agricultural practices.^2^ Particularly, the isolation distance is widely used to ensure the coexistence of GM and non-GM crops in the fields.^3^ These preventive measures are beneficial for keeping the adventitious presence of GM content in non-GM crops below the threshold established by different countries.

Because of the mandatory labeling threshold, the GMO content on non-GMO crops should be estimated to comply with the government regulation. To trace adventitious GM content, numerous studies have explored the pollen-mediated gene flow of GM maize at the field level to model the relationship between the distance and cross-pollination (CP) rate.^4–7^ Maize pollen typically spreads over a limited distance because of its large size, and this relationship is often presented as a fat-tailed distribution. Consequently, leptokurtic distributions more closely describe this relationship.^8^ Some researchers use different pollen dispersal models to predict the number of GM grains among the non-GM maize ears in a field.^9–12^ An efficient and accurate sampling method can provide a confident estimate of GMO content with a small cost for monitoring.

As we known, a suitable sampling scheme is essential for understanding the pollen dispersal tendencies of maize.^7,9^ Furthermore, the required sample size for calculating CP rate within a field can be reduced with the appropriate predictive model and sampling method. Several studies developed different field sampling strategies combined with the dispersal function to predict CP rate for control and monitoring purposes.^7,9,11^ They also compared the accuracy of different sampling methods.

Because CP degree declines rapidly with increasing distance, most sampling schemes operate under the assumption that the material in the recipient fields is heterogeneous. Several studies have found that heterogeneity can be modeled through stratified sampling – dividing different distance zones on the basis of the distance from the pollen source.^12–14^ In addition, as a result of the buffer effect in maize plants, the risk of CP nearby the field borders is higher than in the center field.^12^ Joaquima Messeguer (JM) designed a stratified sampling system based on the distance from the field borders to divide the recipient fields into different zones. Therefore, this stratified sampling system, the so-called JM method, is applied to collect samples to determine the effect of pollen dissemination.^12^ Within-field variability information can also be used as an auxiliary variable to design more efficient sampling strategies. This approach can either reduce the sample size or increase the accuracy of CP rate predictions.^11^

To investigate the suitability of sampling schemes for GM crop monitoring, simple random sampling (SRS) is usually compared with different sampling methods.^7,9,11^ Allnutt et al.^9^ compared four sampling plans with SRS for their ability to predict the GM levels across two transects, four transects, cross transects, and the JM method in several fields at the landscape level. The two-transect sampling had the lowest accuracy and required the smallest sample size, whereas SRS sampling had the highest accuracy and required the largest sample size. The JM method performed as well as SRS. Although SRS was the most accurate method for predicting CP degree, it is too inefficient in the real-world settings. By contrast, systematic random sampling (SYS) is easily performed, and its applicability and reliability were validated in a real-world situation of crop coexistence.^7^ Notably, because small differences in the location of samples can cause large differences in CP rate, the selection of sampling point should not be changed.^9^

Most relevant studies have been implemented in large-scale farming systems; to our knowledge, none have been conducted on the smallholder farming system in Asia, which is characterized by fragmented landscapes and spatial heterogeneity in the field. A particularly common situation in small-scale agriculture is the separation of crop fields by the field border, an intervening strip of land. Thus, the effect of field border on CP rate should be considered. In addition, to monitor the risk of the spread of maize pollen, sampling methods for smallholder farming systems should be compared on the basis of modeling approach.

In this study, we investigated suitable sampling methods for monitoring the risk of pollen-mediated gene flow from neighboring GM maize fields with fragmented landscapes in Taiwan. Considering the distance and rate of CP and the field border effect, we applied empirical modeling for evaluating the method. These findings can be used as the reference for the other Asian countries with smallholder farming systems, such as Japan, Korea, and the Philippines.

## Materials and Methods

### Field Design

Experiments were conducted at the Tainan District Agricultural Improvement Station (23°47′N, 120°26′E) in 2009 and 2010. For the first crop season in (2009–1), the pollen source was at the south edge, and the pollen recipient was designed to be downwind from the pollen source (Fig. S1a). Fig. S1b, c shows the design of the other two types of field experiments with field borders for the second crop season in (2009–2) and for the first crop season in 2010 (2010–1).^15^ In this study, field borders were designed as a 6.75 m and 7.5 m width unplanted area in 2009–2 and 2010–1 experiments, respectively. The density of plant was about 53,000 plants ha^−1^ in 75 cm row spacing and 25 cm plant spacing.

The sampling method evaluation and the simulation study were performed for the 2009–1 crop season. The 2009–2 and 2010–1 crop season data were used to assess the performance of the candidate sampling methods for monitoring and predicting the spread of maize pollen.

### Crop Variety

Two commercial glutinous maize varieties in Taiwan with different grain colors were selected. The Black Pearl, which has purple grains (purple maize), and the Tainan No. 23, which has white grains (white maize) were used as the pollen source and recipient, respectively.^15^

### Data Collection and CP Rate Calculation

The field was divided into 1640 sampling plots (2.5 × 0.75 m^2^) in the 2009–1 crop season. However, in both the 2009–2 and 2010–1 crop seasons, ears were collected from a smaller sampling plot of 1.25 × 0.75 m^2^. In this study, the sampling plot was treated as the sampling point. The full plots data consisted of ears collected within each sampling plot through the experiment field. This procedure was referred to as the full plots survey in our study. In addition, only the first ear of all plants in each sampling plot was collected to calculate the CP rate of the sampling plot. The actual CP rate of each sampling plot was determined by counting the number of purple grains on the white ears of the pollen recipient as

(1) $$ActualCP=\left[\sum_{i=1}^{n}Ear_{i}/(n\times AVK)\right]$$

where *n* is the number of ears at each sampling plot, Ear_i_ the number of purple grains on the *i*^th^ ear at each sampling plot, and AVK the average grain number of an ear in the field.^15,16^

### Sampling Method Layout

We performed both a case study for the 2009–1 field data and a simulation to compare the performance of commonly used sampling methods. The 2009–2 and 2010–1 field data with field border were used to assess the performance of the candidate sampling methods. The sampling methods included two-transect (T_two_), four-transect (T_four_), six-transect (T_six_), cross-transect (T_cross_), V-transect (T_V_), JM method for low expected flow (JM[L]), JM method for high expected flow (JM[H]), SYS, and SRS.^9,,12,,17–19^ In JM(L), four transects were established to divide the long and short sides into trisection. The sampling plots were set on the transects at distances 0 and 3 m from the field edge, and the junction of two transects. The difference between JM(H) and JM(L) is that JM(H) has additional sampling plots on the transects at a distance of 10 m from the field edge.^12^

### Pollen Dispersal Model

The pollen dispersal model used herein and in our previous study^15^ is

(2) $$CP=P_{0}\times 10^{\left(a\sqrt{FB}+b\sqrt{D}\right)}$$

where *P*_0_ is the average CP rate of all sampled plots in the first row at the edge of the pollen recipient field neighboring the pollen source field, FB the isolation distance from the edge of the source to the recipient field, D the distance (m) from the sampled plot to the edge of the neighboring pollen source field, and a and b the model parameters.

### Statistical Analysis

To further evaluate their performance and suitability for monitoring and prediction, the sampling schemes were implemented with 1,000 bootstrap samples and the empirical pollen dispersal models were fitted for samples. The raw full plots data were first resampled with replacement to generate bootstrap full plots data, and then each sampling scheme was implemented on the bootstrap full plots data to produce the bootstrap samples of each sampling scheme. The 95% confidence intervals (CIs) of the root mean square error (RMSE) of the sampling plots were then calculated using the percentile bootstrap confidence interval (PBCI) method.^20^ By sorting the evaluation criteria of 1,000 bootstrap samples, the 95% of PBCI was calculated between the 2.5th and 97.5th percentiles as follows:

$$\left({{\hat{\theta }}^{*}}_{\left(2.5\right)},{{\hat{\theta }}^{*}}_{\left(97.5\right)}\right)$$

Box–Cox plots of average predicted CP rates for the 1,000 samples were used to assess the predicted ability of the sampled data. Evaluation criteria based on the pollen dispersion model used to select the optimal sampling scheme included RMSE, the correlation coefficient r, and relative error. After fitting the pollen dispersal model, the criteria were calculated by comparing the predicted CP value and actual CP of the raw full plots data. All statistical analyses were conducted using SAS (version 9.3; SAS Institute, Cary, NC, USA).

## Results

### Assessment of 2009-1 Field Data

**Figure 1.**
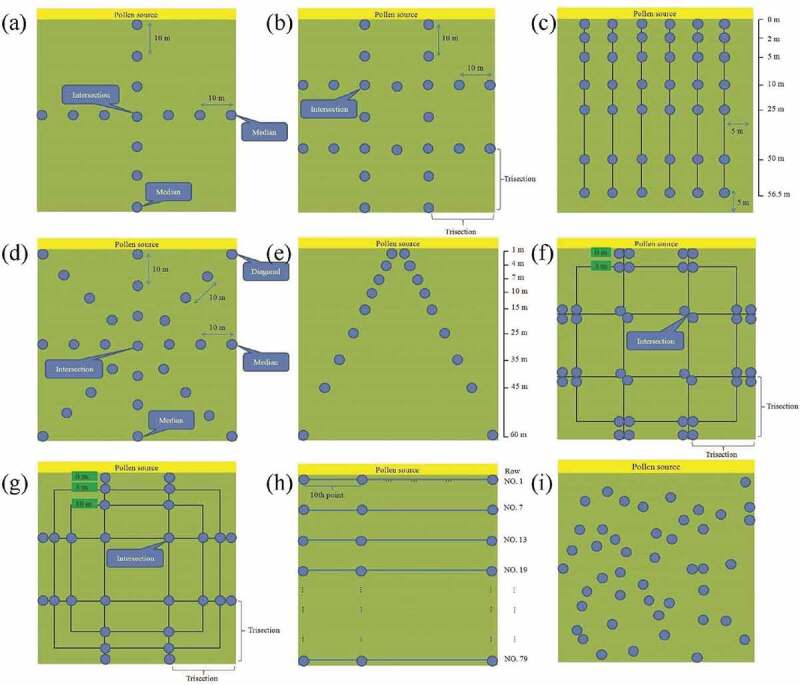
Sampling locations for different sampling methods with the number of sampling plots (n). (a) T_two_ (n = 13); (b) T_four_ (n = 24); (c) T_six_ (n = 42); (d) T_cross_ (n = 29); (e) T_V_ (n = 18); (f) JM(l) (n = 40); (g) JM(h) (n = 28); (h) SYS (n = 42); (i) SRS (n = 322)

Figure 1 illustrates the locations and numbers for different sampling methods of sampling plots (n) for the recipient field in the 2009–1 crop season. The estimates for model parameters a and b from the raw full plots data were 0.6261 and −0.6784, respectively. The positive estimate of parameter a indicates that the field border may enhance pollen exchange. In addition, the negative estimate of parameter b suggests that CP rate decreases as the distance from the pollen source increases.

To assess the performance of the candidate sampling methods, evaluation criteria, including RMSE, r, and relative error, were calculated from the observed CP rates from the raw full plots data of 2009–1 experiment and CP rate predicted by the fitted pollen dispersion model (Table 1). For each sampling method, all linear correlation coefficients r between the observed and predicted CP rates were >0.81 (Table 1). SRS performed better than the other sampling methods except full plots survey, yielding the smallest RMSE and relative error values. However, it required larger sample sizes (n = 322), and the execution of sampling plot placement was more complicated than that of the other methods. SYS, T_cross_ and T_two_ had poorer prediction ability, with higher RMSE and relative error, respectively. In addition, T_four_ had the second highest value of relative error (1.7994).

**Table 1.** RMSE, relative error, and r calculated by the actual CP rate from the full plots survey of 2009–1 experiment and the predicted CP rate for sampled plots using different sampling methods

Methods	n	RMSE	r	Relative error
Full plots survey	1640	0.0200(1) ^†^	0.8566	0.9306 ± 0.0226^‡^(1) ^†^
T_two_	13(1)	0.0248	0.8144	2.2748 ± 0.1186
T_four_	24(3)	0.0223	0.8112	1.7994 ± 0.0928
T_six_	42	0.0206(3)	0.8463	0.9819 ± 0.0360(3)
T_cross_	29(5)	0.0312	0.8544	1.1690 ± 0.0502
T_V_	18(2)	0.0220	0.8485	1.1066 ± 0.0471(5)
JM (L)	40	0.0207(4)	0.8439	1.0968 ± 0.0472(4)
JM (H)	28(4)	0.0215(5)	0.8333	1.1148 ± 0.0498
SYS	42	0.0344	0.8546	1.2091 ± 0.0535
SRS	322	0.0206(2)	0.8486	0.9510 ± 0.0317(2)

^†^Sorting the values of the RMSE, relative error, and sample sizes from smallest to largest, the first five numbers are denoted by (1)–(5).

^‡^Mean ± standard deviation.

RMSE: Root-mean-square-error.

r: Correlation coefficient.

**Figure 2.**
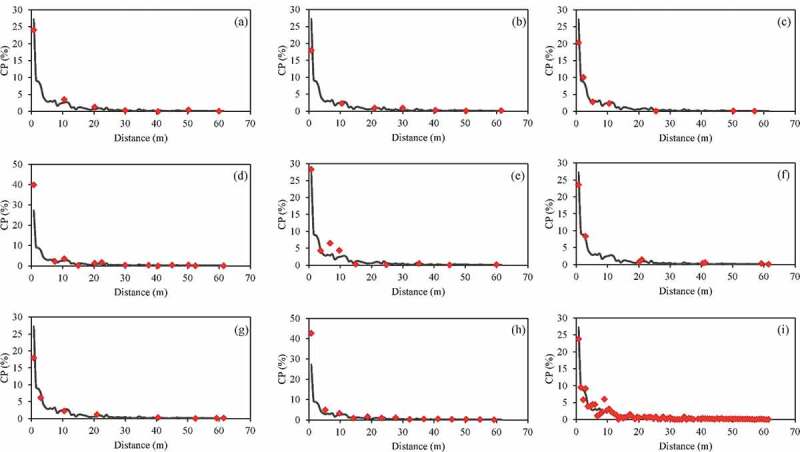
The average CP rate of full plots data (gray line) and different sampling methods (red mark) at each sampled distance from the source. (a) T_two_; (b) T_four_; (c) T_six_; (d) T_cross_; (e) T_V_; (f) JM(l); (g) JM(h); (h) SYS; (i) SRS

Figure 2 presents the average CP rate of full plots data and different sampling methods at different sampling distances from the pollen source. Average CP rate declined with increasing distance from the pollen source in all methods. Furthermore, CP rates from the SRS, JM(L), JM(H), and SYS data were similar to those calculated from the full plots data. The average CP rate declined rapidly across the first 12 m and approached zero at distances over 30 m.

### Bootstrap Simulation

To evaluate the stability of each sampling method in CP rate prediction, the raw 2009–1 full plots data were then used to construct bootstrap samples. After fitting the data from the 1,000 bootstrap samples, the mean and standard deviation of the RMSE and relative error were calculated for the full plots survey and different sampling methods. The T_six_ method performed better than the other sampling methods, with the smallest values of mean and standard deviation of the RMSE (Table 2). The mean and standard deviation of the relative error of the SRS method were smaller than those of the other sampling methods, excepting those of the full plots survey.

**Table 2.** RMSE and relative error calculated by the fitted pollen dispersal model for each method in the simulation analysis

Methods	RMSE^†^	Relative error^†^
Full plots survey	0.0193 ± 0.0014^‡^ (1)	0.9324 ± 0.0006 (1)
T_two_	0.0270 ± 0.0066	1.9404 ± 0.0259
T_four_	0.0255 ± 0.0046	1.8896 ± 0.0177
T_six_	0.0201 ± 0.0017 (2)	1.3616 ± 0.0201
T_cross_	0.0236 ± 0.0038	1.4782 ± 0.0136
T_V_	0.0213 ± 0.0027	1.0355 ± 0.0077 (4)
JM (L)	0.0208 ± 0.0021 (4)	1.0435 ± 0.0073 (5)
JM (H)	0.0220 ± 0.0029	1.1729 ± 0.0120
SYS	0.0210 ± 0.0027 (5)	0.9979 ± 0.0034 (3)
SRS	0.0202 ± 0.0019 (3)	0.9726 ± 0.0027 (2)

^†^Ordered from smallest to largest, the five values of RMSE and relative error are denoted by (1)–(5), respectively.

^‡^Mean ± standard deviation.

RMSE: Root-mean-square-error.

**Figure 3.**
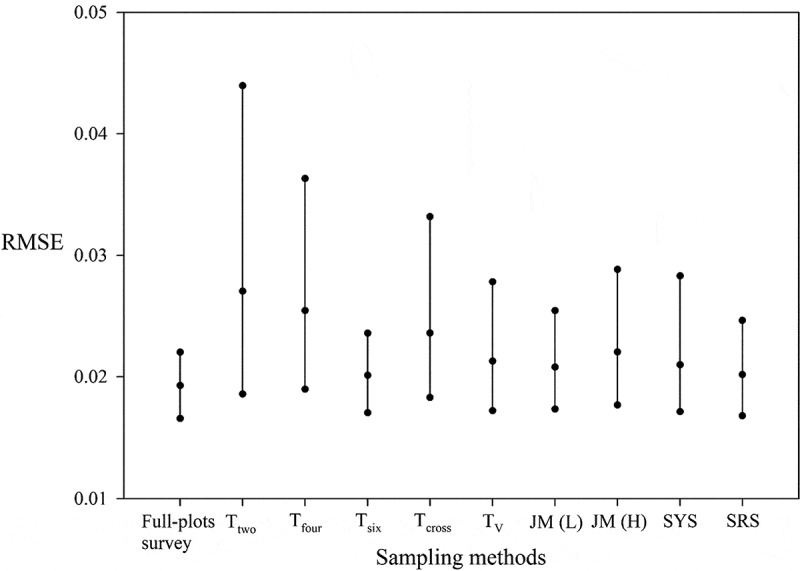
The 95% PBCIs of RMSE calculated for the sampled plots of each method in the simulation analysis

To evaluate the stability of each sampling method in modeling fitting, the CIs of RMSE from the 1,000 bootstrap samples were determined (Fig. 3). The T_six_ and T_two_ methods had the smallest and greatest 95% PBCIs, respectively. Full plots survey showed minimal overlapping of the 95% PBCIs of the T_two_, T_four_, and T_cross_ methods. The upper bounds of these intervals were greater than those of the other sampling methods. However, considerable overlapping of the 95% PBCIs from the full plots survey with those of the other sampling methods was observed. Differences among the methods in the lower bounds of the 95% PBCIs were not substantial. As Fig. 3 shows, the T_six_, T_V_, JM(L), JM(H), SYS, and SRS methods performed close to the full plots survey in estimating the pollen dispersal tendencies of maize through empirical modeling.

**Figure 4.**
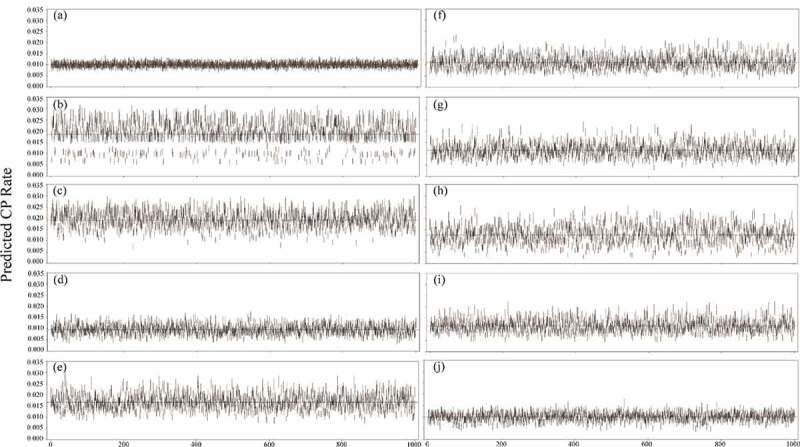
The 95% CIs of the predicted CP rate calculated for the sampled plots at different distances of each method in the simulation analysis: (a) full plots survey, (b) T_two_, (c) T_four_, (d) T_six_, (e) T_cross_, (f) T_V_, (g) JM (l), (h) JM (H), (i) SYS, and (j) SRS. Dashed lines indicate the average CP rate in simulation data of each method

To evaluate the stability of the average predicted CP rate of the whole field, Fig. 4 presents the 95% CIs for each method of the average predicted CP rate of the whole field calculated for the sampling plots at different distances in the simulation analysis. For the T_six_, T_V_, JM(L), SYS, and SRS methods, most CIs were stable, with a small width and less variation. The best CI coverage of the average CP rate (98.1%) was obtained with full plots survey. The five methods with the best CI coverage were SRS (72.0%), T_six_ (63.1%), SYS (48.2%), T_V_ (47.6%), and JM(L) (42.8%). The other sampling methods had a CI coverage of <30%, indicating that these sampling methods are less suitable for predicting pollen spread across the entire maize field. In addition, the T_two_, T_four_, and T_cross_ methods overestimated the pollen dispersal tendencies. Results suggest that the samples from the T_six_, T_V_, JM(L), SYS, and SRS methods could be used to construct a stable empirical model for predicting pollen spread across the entire maize field.

**Figure 5.**
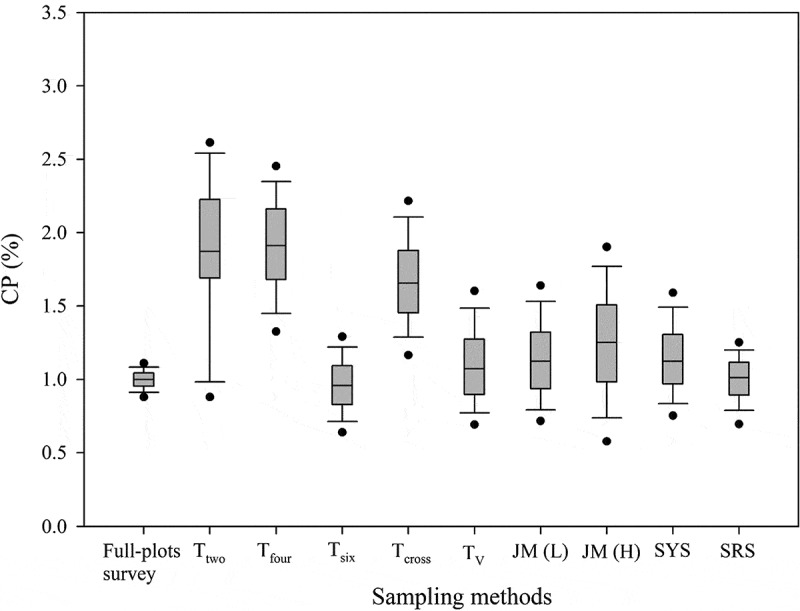
The box plots of the average predicted CP rate for bootstrap runs using different methods

Figure 5 displays the box plots of the average predicted CP rate across the entire fields for each bootstrap run using different methods. The T_six_, T_V_, JM(L), SYS, and SRS methods were stable, with a smaller interquartile range (IQR). By contrast, the T_two_, T_four_, T_cross_, and JM(H) methods were unstable, with larger IQRs. Moreover, the T_two_, T_four_, and T_cross_ methods were clear overestimations. The T_six_, T_V_, JM(L), and SYS methods performed comparably to SRS in predicting the pollen dispersal tendencies through empirical modeling.

According to the bootstrap simulation results, for the predictive ability of pollen dispersal tendencies with the empirical modeling approach, the T_six_, T_V_, JM(L), SYS, and SRS performed better than the other sampling methods except full plots survey. The T_six_, T_V_, JM(L), and SYS methods were easily implemented because their sampling plots were fixed. In their 2008 study, Allnutt et al.^9^ Observed that small differences in sampling location, particularly near field edges, may lead to large differences in CP rate. To prevent subjective bias by field workers, careful and accurate sampling plot selection is crucial. For SYS and SRS, the required sample size depends on the population size of the entire field, which is directly correlated with cost and workload. Therefore, the T_six_, T_V_, and JM(L) methods are recommended for predicting the pollen dispersal tendencies through empirical modeling – particularly the JM(L) method because its sampling plot selection can be performed according to the field shape.

### Actual Field Verification of Performance and Predictive Ability

To evaluate the actual field performance of the T_six_, T_V_, JM(L), SYS, and SRS sampling methods, the data from the 2009–2 and 2010–1 experiments, which involved two field border types, were used. For actual field verification of their predictive ability of the empirical modeling approach, the sampling methods were compared in terms of RMSE, r, and relative error calculated from the full plots data of 2009–2 and 2010–1 experiments.

**Figure 6.**
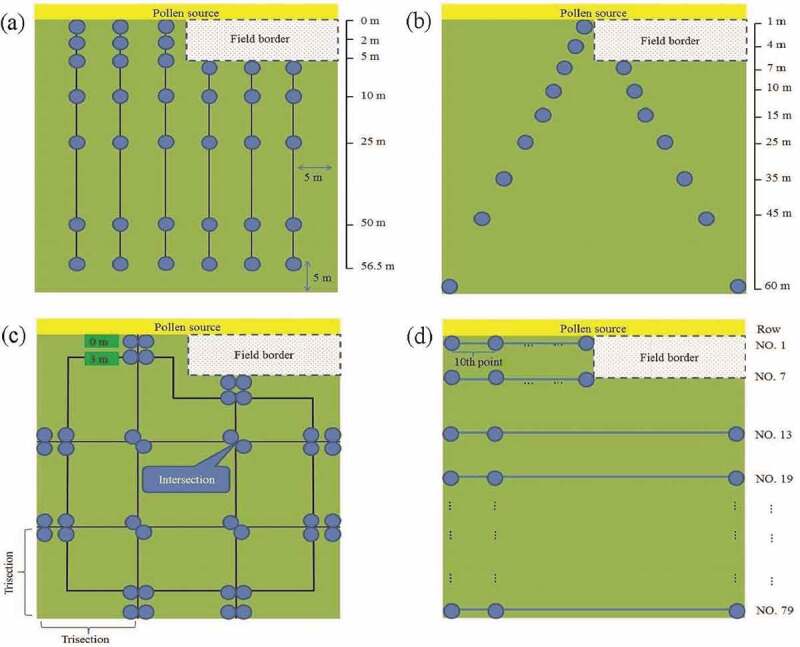
Sampling design of the 2009–2 experimental field. (a) T_six_, (b) T_V_, (c) JM(l), and (d) SYS

The 2009–2 experimental field was designed to simultaneously evaluate the difference between the use and nonuse of a field border. Figure 6 shows the sampling locations. After constructing the empirical model for each method, the RMSE, r, and relative error were calculated (Table 3). The SYS sample had the best predictive ability and the smallest RMSE. The RMSE order of the remaining methods was SRS < T_V_ < T_six_ < JM(L). Excepting that of the SRS, the sample size of the SYS method was greater than that of the other methods. The methods had the same tendency as RMSE for r performance. The small mean and standard deviation of the relative error of the samples indicated that SYS was the most suitable method for predicting pollen dispersal tendencies. The relative error order of the remaining methods was SRS < T_six_ < T_V_ < JM(L). By RMSE, r, and relative error, SYS provided empirical modeling results that were similar to those of the full plots survey, and because its sampling plots were distributed throughout the field, it was convenient to implement.

**Table 3.** RMSE, relative error, r, and sample size in the 2009–2 and 2010–1 experiments for fitting the pollen dispersal model of each method

	RMSE		r		Relative error		Sample size	
Methods	2009–2	2010–1	2009–2	2010–1	2009–2	2010–1	2009–2	2010–1
Full plots survey	0.0322	0.0193	0.9288	0.8328	0.8765 ± 0.0161^†^	1.0124 ± 0.0179^†^	3120^§^	3640^§^
T_six_	0.0383	0.0204	0.9020	0.8324	0.9264 ± 0.0125	0.9820 ± 0.0108	42	42
T_V_	0.0359	0.0225	0.9146	0.7931	0.9662 ± 0.0275	1.3251 ± 0.0528	18	18
JM (L)	0.0432	0.0264	0.8726	0.7761	0.9764 ± 0.0190	1.6215 ± 0.0746	40	40
SYS	0.0321	0.0239	0.9351	0.8258	0.8637 ± 0.0109	1.1572 ± 0.0376	70	76
SRS	0.0324	0.0195	0.9257	0.8306	0.8741 ± 0.0185	1.0153 ± 0.0195	355	361

^†^Mean ± standard deviation.

^§^Total sample size.

RMSE: Root-mean-square-error.

r: Correlation coefficient.

**Figure 7.**
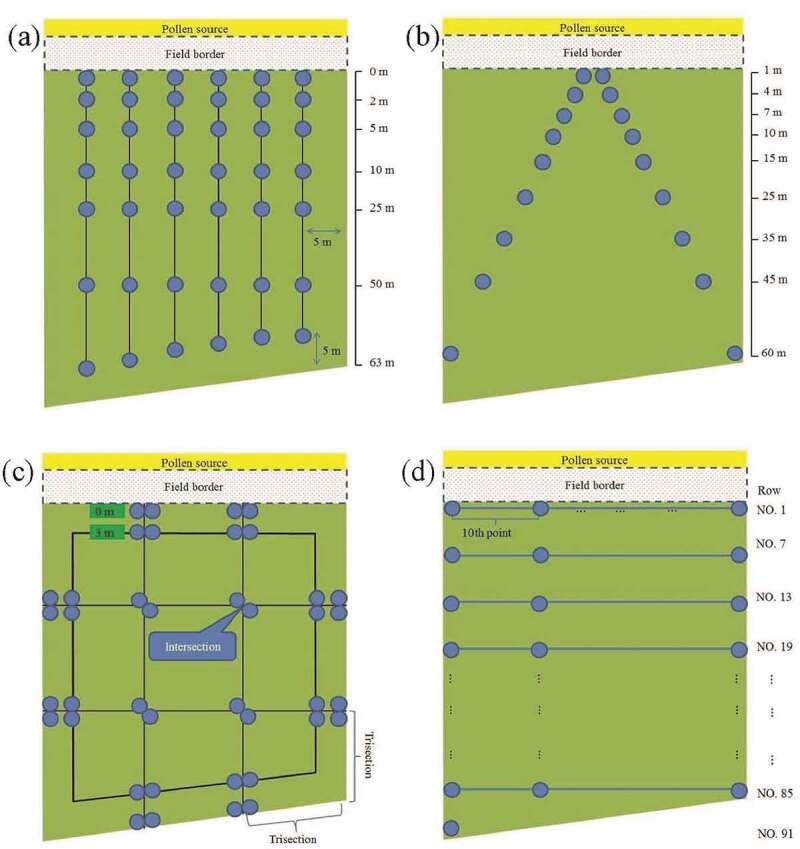
Sampling design of the 2010–1 experimental field. (a) T_six_, (b) T_V_, (c) JM(l), and (d) SYS

The 2010–1 experimental field was also designed to evaluate the field border effect common in the smallholder farming system in Asia. Figure 7 shows the sampling locations. SRS had the best predictive ability and the smallest RMSE value. The RMSE order of the remaining methods was T_six_ < T_V_ < SYS < JM(L). The empirical model fitted to the T_six_ data had the highest mean and standard deviation of the relative error. The order of the remaining methods was SRS < SYS < T_V_ < JM(L) (Table 3). As mentioned, the empirical models fitted to SRS and T_six_ data were the most suitable for predicting pollen dispersal tendencies, with the smallest RMSE and relative error. Compared with that of SRS, the sample size of the T_six_ method was smaller. In addition, T_six_ was easy to implement because its sampling plot locations were fixed.

## Discussion

Suitable sampling methods and empirical pollen dispersal models are necessary for the monitoring and prediction of adventitious transgene presence in non-GM fields.^7^ Robust universal functions and probability distributions for CP rate and distance can be established from the large amount of available data from various field experiments.^9^ In the present study, the pollen-mediated gene flow model, with the consideration of the field border effect, was used to predict CP rate. The CP rate prediction is simplified because of the application of the empirical modeling approach based on distance and field border in our study. The correlation coefficients between the predicted and actual observed values ranged from 0.7761 to 0.9351 in 2009–2 and 2010–1 crop seasons. It means that the pollen dispersal function proposed in our study can be used to compare the effectiveness of different sampling schemes.

For all sampling methods, the relationship between average CP rate and distance was leptokurtic in our study which was consistent with findings from previous studies.^7–9^ Wang et al.^19^ collected samples from V-shaped transects to test a hypothesis of gene flow overestimation and found that pollen deposition decreased exponentially with distance and that the variation of pollen deposition with distance was very small past 50 m. Henry et al.^17^ collected samples from three and six transects in 55 sites across 15 England counties. CP rate rapidly decreased within the first 20 m from the donor crop; beyond this distance, the rate of decrease was considerably slower. In the present study, the average CP rate rapidly declined across the first 12 m and approached zero at distances over 30 m. In several studies that used the SYS^14,18^ or JM sampling methods^12^, the level of adventitious GM content in non-GMO crops was below the European Union’s maximum labeling threshold of 0.9% at a distance of 20 m from the adjacent pollen source field. The average CP rate can also be maintained at <0.9% by using systematic stratified sampling methods by planting 20 m of conventional maize as a pollen barrier between adjacent fields.^21^ Other studies reported larger distances of 30–50 m were required to maintain the GM content at a very low level.^12^ These results were consistent with our finding that CP was almost nondetectable beyond 30 m.

Allnutt et al.^9^ compared the performance of different sampling methods in estimating the GM content in non-GM maize in Spain. The authors found, as we did, that the best approach is SRS but that the large sample sizes required limit its field application. In the present study, the 2009–1 field data also showed that SRS had smaller values of RMSE and relative error but required larger sample sizes (n = 322). However, the T_six_, JM(L), JM(H), and T_V_ methods performed comparably to SRS in CP rate predictions using ≤13% of the SRS sample size.

Various SYS schemes have been proposed and compared with SRS.^7^ Although SYS may yield biased results, it is associated with easier implementation and less risk of subjectivity bias by the field workers than SRS. In the present study, after verification of the 2009–2 and 2010–1 field data, SYS requires a smaller sample size and performed comparably to SRS in predicting the CP rate. The feasibility of sampling schemes is determined from their degree of complexity, sample size, sampling finishing time, and walking distance in the field.^7^ Compared with different SYS schemes, SRS is the most accurate, with the lowest estimated relative error.^7^ However, SRS requires many samples for inspectors to collect, whereas sampling point location is systematic under SYS. Thus, the execution feasibility of SYS is greater than that of SRS. The simplification of the sample selection process can improve efficiency in field studies. The sampling point locations for the T_six_, T_V_, and JM(L) methods are also fixed, making their execution feasibility better than that of SRS as well.

Transect sampling methods are prone to alignment with the gradient of adventitious GM presence in the fields adjoining GM source fields.^9^ Therefore, the JM(L) and JM(H) methods, which operate under a stratified sampling system, are recommended. In the present study, JM(L) predicted the pollen dispersal tendencies more accurately than JM(H). It also performed well across different field shapes in the 2009–2 and 2010–1 experiments. In conclusion, the T_six_, T_V_, JM(L), and SYS methods performed comparably to SRS in CP rate prediction. Because the spatial data may violate the assumption of the ordinary bootstrap method that observations are independent, this may be a limitation for our study. Nevertheless, for more accurate predictions of CP rate in agroecosystems, empirical models in the future probably require more variables to precisely describe the environmental factors on the basis of the selected sampling methods, but some justifications should be needed.

## Supplementary Material

Supplemental Material
