# Supplementary material for: Effectiveness of different sampling schemes in predicting adventitious genetically modified maize content in a smallholder farming system
Source: GM Crops Food. 2020 Dec 10;12(1):212–23. doi: 10.1080/21645698.2020.1846483 (PMC7808422; doi:10.1080/21645698.2020.1846483)
Supplement: Supplemental Material [file KGMC_A_1846483_SM2040.docx]

**Figure Legends**

**Figure S1.** Field design of the experiments for each crop season: (*a*) 2009-1; (*b*) 2009-2; and (*c*) 2010-1. The solid lines indicate the pollen recipient, the dashed lines indicate the pollen source, and the dotted regions indicate the field border.


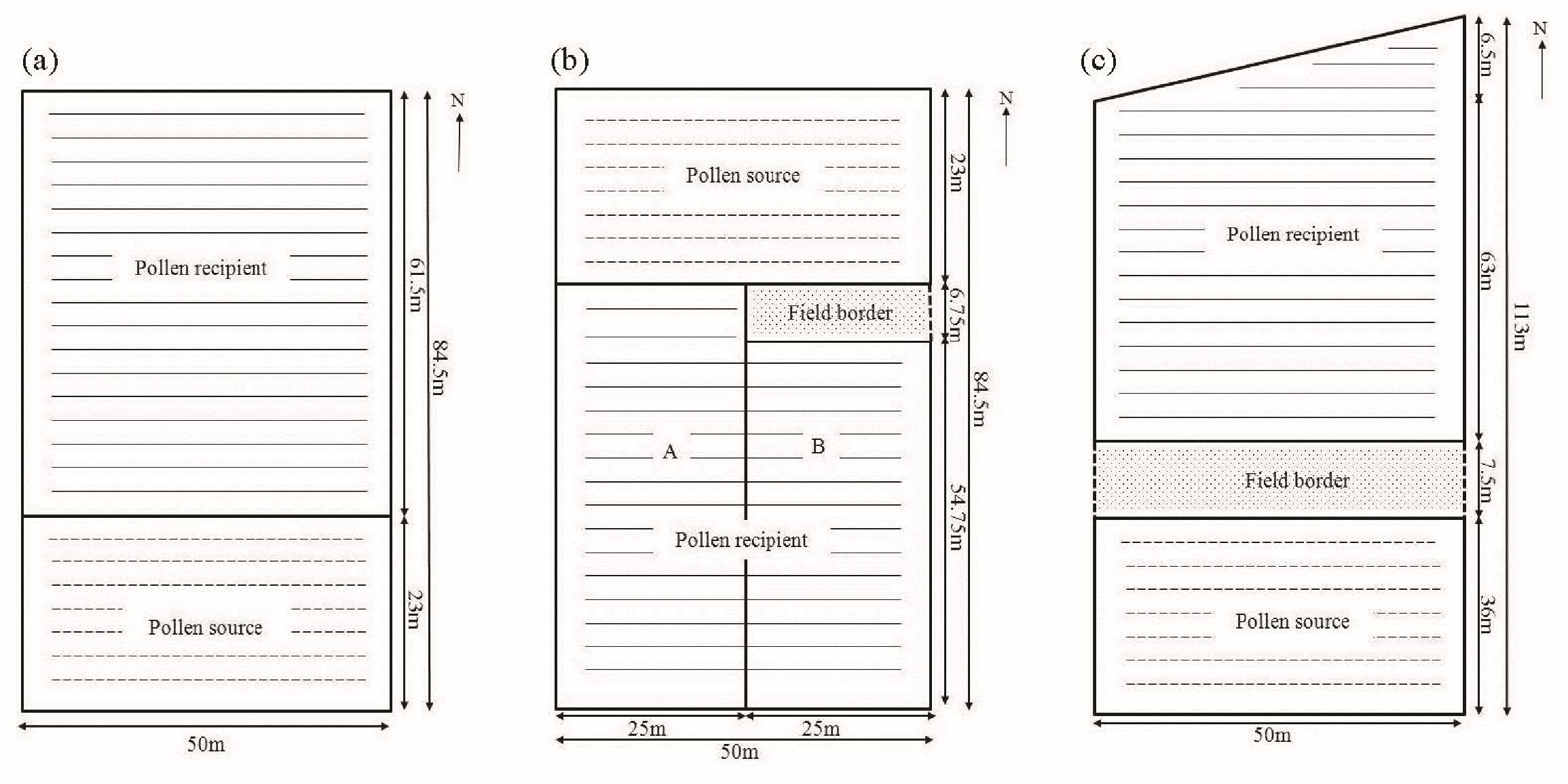


Figure S1
